# A Pharmaco‐Epidemiological Research Program Leveraging a Nationwide Database of Kidney Transplantation

**DOI:** 10.1002/pds.70442

**Published:** 2026-07-23

**Authors:** Pierre Marquet, Antoine Humeau, Sabrina Crépin, Clément Benoist, Sylvain Couderc, Caroline Monchaud, Marc Labriffe, Franck Saint‐Marcoux

**Affiliations:** ^1^ Pharmacology & Transplantation, Université de Limoges, UMR1248 INSERM Limoges France; ^2^ Department of Pharmacology Toxicology and Pharmacovigilance, CHU Limoges Limoges France

**Keywords:** disease modeling, immunosuppressive drugs, kidney transplantation, model‐informed precision dosing, pharmacoepidemiology, real‐world data

## Abstract

**Objectives:**

The pharmaco‐epidemiological research program in kidney transplantation (PERP‐KT) aims to evaluate, for the principal maintenance immunosuppressive drugs (MISDs): the influence of model‐informed precision dosing on graft and patient survival; long‐term exposure—effects relationships; and the benefit‐harm balance of their combinations and time sequences in patient groups or clinical settings not adequately evaluated in comparative randomized clinical trials. It also aims to develop a hybrid, dynamic, deep learning model capable of predicting the rate of renal graft function decline, thereby providing a platform for individualized prediction of the benefits and risks associated with MISDs.

**Patients and Methods:**

After obtaining all regulatory andd ethical approvals, de‐identified extracts of three national databases were linked to create the nationwide PERP‐KT dataset, which is hosted within the highly secure environment of the French Health Data Hub.

**Results:**

CRISTAL (the exhaustive registry of the French Agence de la Biomédecine) comprises data from 49 886 kidney donors and the corresponding 47 842 transplant recipients between 2005 and 2020. Following iterative deterministic matching and extensive quality control procedures, CRISTAL data were successfully linked to: the French national Health Data System (SNDS), which records reimbursed healthcare utilization, for 30 782 kidney transplant recipients; and to ISBA, a web‐based platform for Bayesian dose adjustment of MISDs that contains pharmacological data, for 17 700 kidney grafts and 17 576 recipients.

**Perspectives:**

More than 20 pharmaco‐epidemiological studies will leverage the database's extensive follow‐up, large population size and richness of clinical, healtcare‐utilization, and pharmacological data.

## Keypoints

- An unprecedented nationwide, longitudinal database in kidney transplantation has been gathered to support a vast pharmaco‐epidemiological research program.
- It contains clinical data about the donor and the recipient for about 50 000 transplants, the exhaustive list of drugs delivered and healthcare provided for 64% of them, and detailed pharmacological data about maintenance immunosuppressive drugs for 37%.
- Patients were followed up for up to 17 years after transplantation.
- These exceptional population sizes, time‐depth, and wealth of clinical information will support more than 20 pharmaco‐epidemiological studies.

## Plain Language Summary

This research program on kidney transplantation (PERP‐KT) intends to evaluate: the efficacy of fine‐tuned, individual dose adjustment of the main maintenance immunosuppressive drugs (MISDs) on the longevity of the graft and on patient survival; the long term effects of different blood levels of the main MISDs; the efficacy and adverse effects of the different combinations and time sequences of MISDs in patient groups or clinical conditions not previously evaluated; and to develop an artificial intelligence tool able to predict the speed at which each kidney graft will deteriorate after transplantation. PERP‐KT leverages the CRISTAL registry (managed by the French Agence de la Biomédecine) exhaustively listing kidney transplant procedures in France (49 886 donors and 47 842 recipients between 2005 and 2020), combined with the SNDS (Système National des Données de Santé) that contains all drugs delivered and healthcare activities for 30 782 of them, and ISBA (ImmunoSuppressive Bayesian dose Adjustment website) a database about the dose and blood levels of the main MISDs for 17 700 kidney grafts and 17 576 patients. This unprecedented database will support more than 20 studies on the efficacy and adverse events of MISDs and will foster personalized medicine in kidney transplantation.

## 1 Introduction

After kidney transplantation, life‐long therapy with maintenance immunosuppressive drugs (MISDs) is necessary to avoid severe graft rejection and graft loss‐of‐function. Kidney transplantation offers patients a longer life expectancy than dialysis, but despite anti‐rejection treatment, it is still shorter than in the general population. Graft survival is approx. 14 years on average in France [1]. Consequently, an increasing proportion of patients are registered repeatedly on the national kidney transplant waiting list, prolonging the time on waiting lists for all. The MISDs available for long‐term maintenance treatment (cyclosporine, tacrolimus, mycophenolic acid, everolimus) were approved in the late 1990's or before, except for belatacept, approved by the EMA in 2011 but underused for a long time due to limited availability. These drugs are almost always used in combinations, possibly evolving after serious rejection episodes, adverse drug reactions or comorbidities, resulting in different treatment sequences. Most, if not all, are also dose adjusted based on blood levels. The level of evidence of the best treatment strategies is often limited to expert opinions. This is because many clinical trials had a limited duration of 1–3 years and were performed in the late 1990's when patients were essentially younger and with less comorbidities than now. Also, not all drug combinations have been compared, and second‐line treatments after serious comorbidities, rejection episodes or adverse drug reactions have rarely been tested. As a result, clinical and therapeutic practices vary largely between transplantation centers, globally as well as in France, and over time. Despite these limitations, current treatments with drugs in use for 30 years or more are very efficient overall, to the point that about a dozen candidate MISDs have failed to demonstrate a better efficacy‐safety profile in registration trials. One possible way to improve immunosuppressive treatment harm‐benefit balance is to identify for each type of patient profile, clinical condition, or even each patient, the best first‐, second‐ or third‐line MISDs combinations, the best doses and/or dose adjustment strategies for certain or each of the MISDs. This is one of the often‐overlooked modalities of precision medicine. In this context, Artificial Intelligence (AI) increases researchers' capabilities to extract information on treatment effects from real‐world data, which can then be accounted for by patient care guidelines.

**FIGURE 1:**
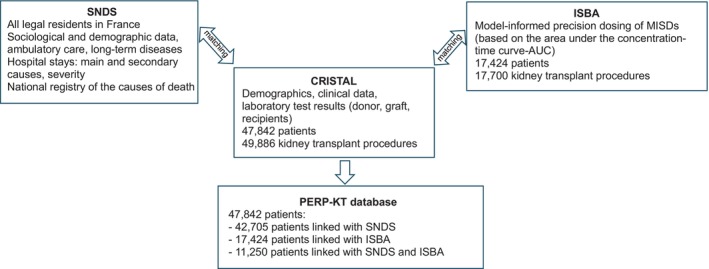
Flowchart of database linkage.

The pharmaco‐epidemiological research program in kidney transplantation (PERP‐KT) leverages a nationwide database hosted by the French government‐driven Health Data Hub (HDH). The database is made up of de‐identified (“pseudonymized”) extracts from three national databases, CRISTAL (Agence de la Biomédecine, ABM), Système National des Données de Santé (SNDS), and ImmunoSuppressive Bayesian dose Adjustment expert system (ISBA) (Figure 1).

ISBA is made available through a website managed by Limoges University hospital (CHU Limoges) since 2005 and authorized by the French Data Protection Authority (CNIL) under authorization number 1619537, offering MISDs pharmacokinetics‐guided dose adjustments for organ transplant physicians and patients. It has received more than 190 000 requests in 21 years for MISDs individual dose adjustment based on the area under the concentration‐time curve (AUC), from more than 200 transplantation centers spread out on all continents, including many kidney transplant centers in France. However, the database comprising all these pharmacological data (drug dosing, minimum and maximum blood concentrations—*C*_min_/*C*_max_, AUC) could not be used alone because of the lack of clinical context and of “controls,” without pharmacokinetics‐guided dose adjustments. For this reason, we paired it with CRISTAL, the exhaustive registry of all organ transplants in France, for all kidney transplant procedures in France between 1 January 2005 and 31 December 2020. This registry is authorized by CNIL under authorization number 96‐025. CRISTAL contains demographics, clinical data and laboratory test results from the donor, the graft and the recipient (before, at, and yearly after transplantation). The CRISTAL extract used for PERP‐KT was also paired with SNDS, the database listing all healthcare expenses for beneficiaries of the National Health Insurance for employees (CNAMTS) in France. SNDS lists hospital stays (including principal, secondary diagnoses, and severity level), medical consultations, paramedical acts, drug delivery, and comprises the national registry of deaths. Extensive description of the French medico‐administrative databases is available elsewhere [2].

PERP‐KT spans four research lines totalizing more than 20 individual studies, depending on patient age (pediatrics, adults), MISDs and comorbidities. The first aims are to evaluate the influence of AUC‐guided individual dose adjustment (also called model‐informed precision dosing) of the MISDs following an exposed vs. non‐exposed design. The second aims to evaluate the long‐term exposure–effects relationships of the main MISDs, specifically regarding *C*_max_, *C*_min_ and AUC. The third theme will consist in evaluating the benefit–risk balance of the combinations and sequences of MISDs in patient groups or circumstances not evaluated as part of comparative randomized clinical trials, following a case–control design nested in the cohort. The last objective is to develop longitudinal (time‐dependent), deep learning models able to forecast the velocity of renal graft function deterioration (VGFD) post‐transplantation, as a platform to predict long‐term MISDs effects at the population and individual levels.

## 2 Patients and Methods

### 2.1 Ethics and Regulatory Approval, Data Security Management

The HDH offers a secured space, where users can access and analyze data in conformity with the security measures applicable to SNDS and its own “general conditions of use” and “security policy of HDH information system.” The research activities performed in the PERP‐KT secured space are under the responsibility of CHU Limoges, which homologated this research space after “data protection impact analysis” and “informatics risk analysis.” CHU Limoges and HDH have gathered all data documentation in a unique file exhaustively describing the extracted data (available upon request). The database can be used for no purpose other than those described in the initial research program and approved by CNIL, on 20 November 2020 (authorization number 920315) following approval by the national ethics committee for health data research (CEREES). Due to the population size and time depth, CNIL approval included the authorization of informing patients through their transplant physicians and the CHU Limoges and HDH websites, rather than individually. This study is registered by the HDH as T11068872019112.

### 2.2 Secondary Use of Data

The PERP‐KT dataset is not publicly available, and individual‐level data cannot be directly transferred to external investigators. Besides, Agence de la Biomédecine did not approve reuse of the CRISTAL registry extract for any other purpose or investigators than those of PERP‐KT. Future research may still use the linked SNDS‐ISBA dataset, pending scientific review, approval by the relevant data controllers, compliance with data minimization principles, and adherence to the applicable HDH, SNDS, CHU Limoges, and CNIL requirements. Access to individual‐level data remains restricted to the secured HDH environment and authorized users.

The SNDS‐ISBA population is made of the same patients as the CRISTAL‐SNDS‐ISBA subgroup detailed in Table 3 and Figure 1, however, there is no information regarding the donor, the pre‐transplant period, the transplant procedure, the induction immunosuppressive regimen, and early complications post‐transplant. In contrast, maintenance immunosuppression and all other community‐delivered treatments, all hospital stays with their main and secondary causes, all medical visits, as well as the national registry of deaths are included in the SNDS. In addition, although CRISTAL has been so far the only reliable source of graft loss occurrence and timing in PERP‐KT, the HDH asked for the setting up of an algorithm to infer kidney graft loss from SNDS data, which should be available within a few months.

Independent of PERP‐KT, CRISTAL data may be requested from Agence de la Biomédecine (https://www.agence‐biomedecine.fr/fr/institutionnel/nous‐contacter), SNDS data from Assurance Maladie (https://www.assurance‐maladie.ameli.fr/etudes‐et‐donnees/en‐savoir‐plus‐snds/utilisation‐accompagnement‐donnees‐snds) and ISBA data from Limoges University Hospital (https://www.chu‐limoges.fr/recherche‐et‐innovation/delegation‐a‐la‐recherche‐clinique‐et‐a‐linnovation‐drci/).

### 2.3 Matching ISBA and CRISTAL

All adult and pediatric kidney transplant recipients who underwent transplantation in France between January 2005 and December 2020 were recorded in the national transplant registry, CRISTAL, and were eligible for linkage.

**FIGURE 2:**
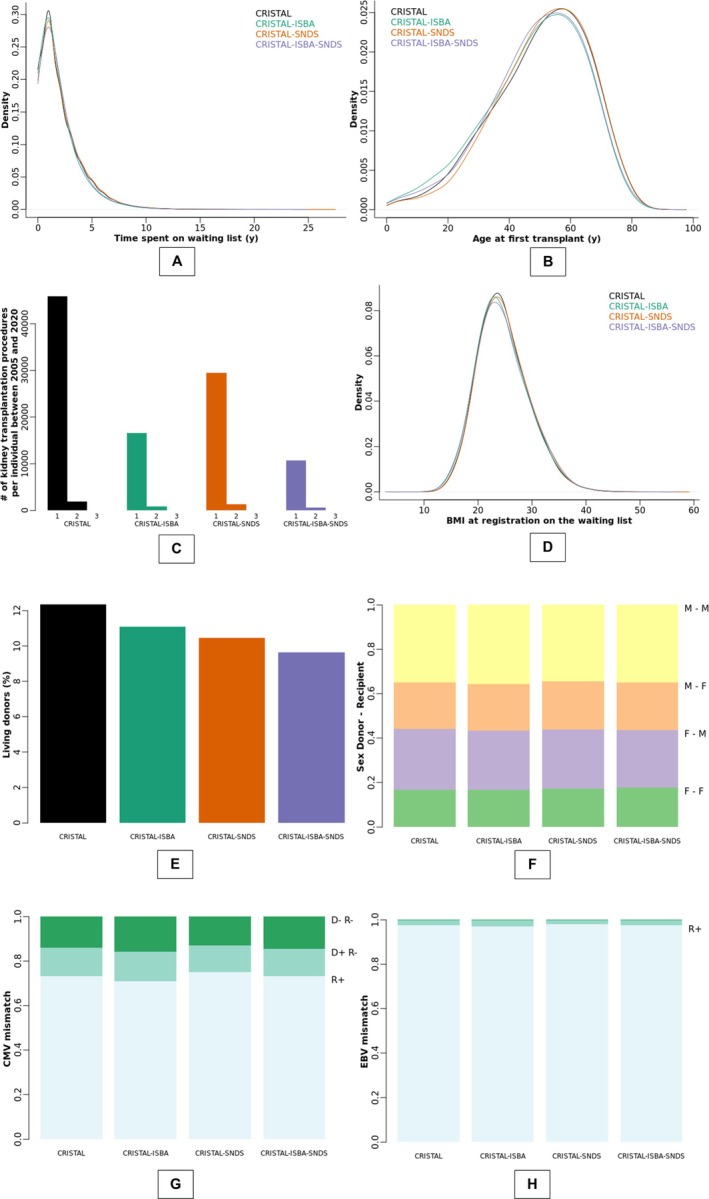
Comparative descriptors of the different study populations resulting from the linkage of the three original databases (the ISBA‐SNDS population is identical to the CRISTAL‐ISBA‐SNDS and is not shown). (A) Distribution of time spent on the waiting list. (B) Distribution of age at first transplantation. (C) Number of first, second or third (and more) transplantation procedures per patient. (D) Distribution of body mass index (BMI) at transplantation (all procedures). (E) Percentage of living donors. (F) Donor/Recipient sex matching. (G) Donor/Recipient CMV status at transplantation (all procedures). (H) Donor/Recipient EBV status at transplantation (all procedures).

CRISTAL and ISBA matching pointed to a sub‐cohort of patients who underwent pharmacokinetic‐guided immunosuppressive drug dose adjustments (exposed), who can then be compared with patients recorded in CRISTAL but not found in ISBA, considered here as controls (Figure 2).

The following variables were used by the ABM to link the CRISTAL and ISBA datasets following “iterative deterministic patient matching” [3]: birth date; transplantation date; name (full name in CRISTAL, only initials in ISBA, sometimes with inconsistencies between occasions); transplant and follow‐up centers.

The iterative deterministic matching algorithm consisted of 20 matching rules with progressively relaxed criteria (Table 1). CRISTAL patients sometimes matched several ISBA patients because of less reliable patient identification in ISBA.

**TABLE 1:** Matching ISBA and CRISTAL: Multistep database matching rules.

Successive matching rules	Names	Birthdate	Transplantation (Tx) date	Centers
Rule 1	First and last name	Birthdate	Tx date	
Rule 2	First and last name	Birthdate	Tx date ± 15 days	
Rule 3	First and last name	Birthdate		Tx/follow‐up Center
Rule 4	First and last name	Birthdate	Tx date/1st tx date	
Rule 5	Alternative first and last name^a^	Birthdate	Tx date	
Rule 6	Alternative first and last name	Birthdate	Tx date ± 5 days	
Rule 7	Alternative first and last name	Birthdate		Tx/follow‐up Center
Rule 8	Alternative first and last name	Birthdate	Tx date/1st tx date	
Rule 9	Last name	Birthdate	Tx date	
Rule 10	Last name	Birthdate	Tx date ± 5 days	
Rule 11	Last name	Birthdate		Tx/follow‐up Center
Rule 12	Last name	Birthdate	Tx date/1st tx date	
Rule 13	First and last name	Birthdate	Month and day of tx	
Rule 14	First and last name	Month and day of birth	Tx date	
Rule 15	First and last name	Birthdate	Year and day of tx	
Rule 16	First and last name	Year and day of birth	Tx date	
Rule 17	First and last name	Birthdate	Year and month of tx	
Rule 18	First and last name	Year and month of birth	Tx date	
Rule 19	First name	Birthdate	Tx date	Tx/follow‐up Center
Rule 20	Inversion of first and last name	Birthdate	Tx date	

^a^ Two sets of initials in ISBA were tested because of inconsistencies.

### 2.4 Matching SNDS and CRISTAL

An iterative and deterministic strategy was also employed to link SNDS and CRISTAL, the latter being used as the reference (Figure 2). The matching algorithm, adapted from Auffray et al. [4], was developed in collaboration with the ABM, HDH, and CNAMTS. It consisted of eight matching rules, with progressively relaxed criteria (Table 2). Identifiers common to both databases were utilized in the algorithm: gender, month and year of birth, transplantation center, date of renal transplantation, living/deceased status of the recipient on last follow‐up, date of death, return‐to‐dialysis date, start of renal replacement therapy date, and last known residence postcode. If an identifier in CRISTAL corresponded to a single identifier in the SNDS, or vice versa, data were linked. If not, the identifiers proceeded to the next step in the algorithm. Several codes were used to retrieve patients with renal transplant in the SNDS: ICD‐10, CCAM (French standardized coding system for medical procedures), and GHM (Diagnosis‐Related Groups used in France for the reimbursement of hospital activities) codes for kidney transplant and dialysis (Table S1).

**TABLE 2:** Matching SNDS and CRISTAL: Multistep database matching rules.

Successive matching rules	Matching criteria
Rule 1	Birthdate^a^, sex, transplantation date ± 2 days, geographical FINESS^b^ code, ICD‐10, CCAM and GHM codes for kidney transplantation
Rule 2	Birthdate^a^, sex, transplantation date ± 2 days, legal FINESS code, ICD‐10, CCAM and GHM codes for kidney transplantation
Rule 3	Birthdate^a^, sex, dialysis starting date ± 2 days, geographical FINESS code, ICD‐10, CCAM and GHM codes for dialysis
Rule 4	Birthdate^a^, sex, dialysis starting date ± 2 days, legal FINESS code, ICD‐10, CCAM and GHM codes for dialysis
Rule 5	Birthdate^a^, sex, return to dialysis date ± 2 days, geographical FINESS code, ICD‐10, CCAM and GHM codes for dialysis
Rule 6	Birthdate^a^, sex, return to dialysis date ± 2 days, legal FINESS code, ICD‐10, CCAM and GHM codes for dialysis
Rule 7	Birthdate^a^, sex, death date ± 5 days, last known city/department of residence, death
Rule 8	Birthdate^a^, sex, last known city of residence

^a^ Month and year of birth.

^b^ Fichier National des Établissements Sanitaires et Sociaux.

In France, all hospitals and healthcare centers are identified by two Fichier National des Établissements Sanitaires et Sociaux (FINESS) codes: a geographical code, which refers to the specific physical location of a healthcare facility, and a legal code, which designates the institution as a legal entity. At each step, the transplantation center was initially considered based on its geographical localization and subsequently based on the legal entity hosting it (mostly university hospitals).

Among CRISTAL patients successfully matched to the SNDS, additional eligibility criteria were applied to account for data quality and availability constraints within the SNDS. Specifically, only individuals affiliated with the general scheme of the French National Health Insurance were retained for analyses involving SNDS data. This scheme covers nearly 80% of the French population and provides data that is consistently structured, complete, and continuously available throughout the study period (2006–2021). In contrast, the integration of other insurance schemes (e.g., MSA or RSI, French social security schemes for farmers and agricultural workers or self‐employed workers) into the SNDS was more recent, gradual and sometimes inconsistent, which could introduce bias due to differences in coding systems, healthcare access, or data completeness. This restriction, commonly applied in pharmaco‐epidemiological studies, aims to ensure methodological robustness and consistency across the study period [2]. Beyond the difficulty of accurately linking such individuals during the matching process, twins could not be reliably distinguished within the SNDS, in the hospital discharge data, where their records may sometimes be indistinguishable. Therefore, twins were further excluded for analyses involving SNDS data.

For all patients included in the cohort, all available data was extracted for the period between January 2006 (date where the SNDS data became available) and 31 December 2021. For patients transplanted in 2005, SNDS data is missing over the first months, but for patients transplanted in 2006 onwards, this fixed observation period allowed for the reconstruction of the patients' medical history before and after transplantation.

Several objectives of this research program do not require data from ISBA, whenever drug exposure is not necessary, or from the SNDS when they are reliably informed in CRISTAL (e.g., graft loss). Therefore, the exhaustive CRISTAL data from all recipients of a kidney allograft in France between 1 January 2005 and 31 December 2020, with a follow‐up of at least 1 year (hence data extraction up to 31 December 2021) will be used in this research.

A PERP‐KT scientific committee was set up under the umbrella of the HDH to help the research group with strategy, planning, and result interpretation. It is made up of six nephrologists from six different transplant centers in France, three pharmacologists, two representatives of patient associations, one representative of the HDH, and one of the Biomedicine Agency. The committee meets twice yearly.

## 3 Results

The CRISTAL extract comprises data about 49 886 transplant procedures and the corresponding 47 842 different graft recipients, with up to 17 years follow‐up. The nature and completeness of CRISTAL data are described in Table S2. The structure of the CRISTAL registry has evolved with time, as medical science has, and certain variables of interest, particularly concerning the recipient immune status, have only been collected over the last few years of the PERP‐KT timespan. For instance, only since 2017 for HLA‐DQ incompatibilities.

SNDS and ISBA were independently paired with the CRISTAL extract using an iterative deterministic strategy and all the data for the relevant patients were extracted and stored on the HDH. Among the 18 733 patients in the ISBA database, 17 576 (94%) were successfully matched to CRISTAL as unique entries (Table 3). The first two rules successfully matched 79% of the patients, whereas 1157 (6%) patients from ISBA remained unmatched by the end of the process. CRISTAL and SNDS were successfully matched as unique entries for 42 705 patients (89%) (Table 4). The first two steps of the algorithm successfully matched 70% of the patients. Conversely, some rules retrieved fewer than 2% of all matches (rules 3 to 6). Ultimately, 1775 pairs were not unique matches, and 3357 patients from CRISTAL remained unmatched. Applying the SNDS‐specific eligibility criteria resulted in a definitive number of 30 782 patients with SNDS linked to CRISTAL. Most exclusions were related to the requirement for being registered with the general health insurance scheme (*n* = 11 270) – otherwise reimbursement data is not exhaustively collected. Other exclusion criteria were: unconfirmed sex or birthdate, twins, no transplant procedure identified, no ambulatory healthcare in the year preceding transplant. The differences in sociodemographic characteristics between CRISTAL patients linked to SNDS and those not linked are displayed in Table S3. Table 5 summarizes and compares the characteristics of patients whose CRISTAL dossier could or could not be matched with ISBA, SNDS or both, together with the percent missing data, essentially showing very similar characteristics overall, except for % of living donors (lowest in the fully matched dataset), urological pathologies (highest in the fully matched dataset) and transplant center distribution, with proportions of patients addressed to ISBA varying from 2% to 85% (mean = 42%).

**TABLE 3:** Numbers and cumulative percentages of ISBA patients linked with CRISTAL at each matching step.

Successive matching rules	Number of ISBA patients to be matched	Additional unique matches	Cumulative unique matches		Unmatched patient numbers
			*N*	(%)	
Rule 1	18 733	12 907	12 907	(68.9)	5826
Rule 2	5826	1918	14 825	(79.1)	3908
Rule 3	3908	602	15 427	(82.4)	3306
Rule 4	3306	3	15 430	(82.4)	3303
Rule 5	3303	247	15 677	(83.7)	3056
Rule 6	3056	19	15 696	(83.8)	3037
Rule 7	3037	3	15 699	(83.8)	3034
Rule 8	3034	0	15 699	(83.8)	3034
Rule 9	3034	369	16 068	(85.8)	2665
Rule 10	2665	104	16 172	(86.3)	2561
Rule 11	2561	47	16 219	(86.6)	2514
Rule 12	2514	0	16 219	(86.6)	2514
Rule 13	2514	4	16 223	(86.6)	2510
Rule 14	2510	33	16 256	(86.8)	2477
Rule 15	2477	2	16 258	(86.8)	2475
Rule 16	2475	40	16 298	(87.0)	2435
Rule 17	2435	16	16 314	(87.1)	2419
Rule 18	2419	58	16 372	(87.4)	2361
Rule 19	2361	1204	17 576	(93.8)	1157
Rule 20	1157	0	17 576	(93.8)	1157

**TABLE 4:** Numbers and cumulative percentages of SNDS patients linked with CRISTAL at each matching step.

Successive matching rules	Number of CRISTAL patients to be matched	Additional unique matches	Cumulative unique matches *N* (%)	Unmatched patient numbers
Rule 1	47 842	24 003	24 003 (50.2%)	23 839
Rule 2	23 839	9607	33 610 (70.3%)	14 232
Rule 3	14 232	107	33 717 (70.5%)	14 125
Rule 4	14 125	174	33 891 (70.8%)	13 951
Rule 5	13 951	335	34 226 (71.5%)	13 616
Rule 6	13 616	178	34 404 (71.9%)	13 438
Rule 7	13 438	2635	37 039 (77.4%)	10 803
Rule 8	10 803	5666	42 705 (89.3%)	5137

**TABLE 5:** Comparison of sociodemographic data and comorbidities at inclusion in the different subgroups, depending on the success of database matching.

Patient characteristics^a^, ^b^	CRISTAL database *N* = 47 842	CRISTAL matched with ISBA *N* = 17 424	SMD^c^ vs. no ISBA matching	CRISTAL matched with SNDS *N* = 30 782	SMD vs. no SNDS matching	CRISTAL matched with ISBA and SNDS *N* = 11 250	SMD vs. no ISBA and no SNDS matching
Donor vital status (living donor)	5946 (12.4)	1942 (11.1)	0.06	3248 (10.6)	0.16	1094 (9.7)	0.11
Missing # (%)	1 (0.0)	0 (0.0)		0 (0.0)		0 (0.0)	
Time spent on the waiting list (years)	1.0 [1.0–3.0]	1.0 [1.0–2.0]	0.09	1.0 [1.0–3.0]	0.08	1.0 [1.0–3.0]	0.03
Age at registration on the waiting list (years)	50.0 [38.0–60.0]	49.0 [36.0–59.0]	0.18	50.0 [39.0–60.0]	0.08	49.0 [38.0–59.0]	0.09
Age at first transplantation (years)	52.0 [40.0–62.0]	51.0 [38.0–61.0]	0.19	53.0 [41.0–62.0]	0.10	51.0 [40.0–61.0]	0.10
Age categories (years)			0.22		0.17		0.15
[0–10]	556 (1.2)	310 (1.8)		350 (1.1)		182 (1.6)	
[10–20]	1216 (2.5)	666 (3.8)		612 (2.0)		332 (3.0)	
[20–30]	3368 (7.0)	1337 (7.7)		1793 (5.8)		724 (6.4)	
[30–40]	6148 (12.9)	2367 (13.6)		3962 (12.9)		1536 (13.7)	
[40–50]	9231 (19.3)	3513 (20.2)		6258 (20.3)		2406 (21.4)	
[50–60]	12 134 (25.4)	4395 (25.2)		7892 (25.6)		2869 (25.5)	
[60–70]	10 736 (22.4)	3667 (21.0)		7030 (22.8)		2424 (21.5)	
[70–80]	4204 (8.8)	1125 (6.5)		2728 (8.9)		743 (6.6)	
[80–90]	249 (0.5)	44 (0.3)		157 (0.5)		34 (0.3)	
Sex (male)	30 008 (62.7)	10 861 (62.3)	0.01	18 808 (61.1)	0.09	6833 (60.7)	0.05
BMI at registration on the waiting list (kg/m^2^)	24.2 [21.3–27.6]	24.0 [21.1–27.5]	0.05	24.3 [21.5–27.8]	0.08	24.2 [21.2–27.8]	< 0.01
Missing # (%)	2704 (5.7)	950 (5.5)		1551 (5.0)		*554 (4.9)*	
Comorbidities at registration on the waiting list							
Arrhythmia	1583 (4.5)	544 (4.3)	0.02	1036 (4.4)	0.01	359 (4.2)	0.02
Missing # (%)	12 323 (25.8)	4625 (26.5)		7130 (23.2)		2655 (23.6)	
Coronary insufficiency	2633 (7.4)	876 (6.9)	0.03	1808 (7.7)	0.03	607 (7.1)	0.02
Missing # (%)	12 447 (26.0)	4657 (26.7)		7216 (23.4)		2679 (23.8)	
Heart failure	1461 (4.2)	490 (3.8)	0.02	1007 (4.3)	0.02	336 (3.9)	0.02
Missing # (%)	12 647 (26.4)	4665 (26.8)		7356 (23.9)		2689 (23.9)	
Stroke (ischemic/hemorrhagic)	1340 (3.7)	447 (3.5)	0.02	913 (3.8)	0.01	300 (3.5)	0.02
Missing # (%)	12 066 (25.2)	4565 (26.2)		6968 (22.6)		2620 (23.3)	
Myocardial Infarction	1526 (4.3)	500 (3.9)	0.03	1037 (4.4)	0.01	346 (4.0)	0.02
Missing # (%)	12 158 (25.4)	4569 (26.2)		7029 (22.8)		2628 (23.4)	
Unstable angina pectoris	369 (1.0)	125 (1.0)	0.01	246 (1.0)	< 0.01	86 (1.0)	0.01
Missing # (%)	12 308 (25.7)	4609 (26.5)		7124 (23.1)		2646 (23.5)	
Transient ischemic attack	790 (2.2)	310 (2.4)	0.02	534 (2.3)	0.01	212 (2.5)	0.02
Missing # (%)	12 252 (25.6)	4597 (26.4)		7079 (23.0)		2634 (23.4)	
Hypertension	23 915 (68.1)	9043 (70.7)	0.09	16 221 (69.3)	0.08	6160 (71.9)	0.11
Missing # (%)	12 704 (26.6)	4639 (26.6)		7361 (23.9)		2677 (23.8)	
Diabetes	7290 (19.7)	2346 (17.4)	0.09	5132 (20.8)	0.09	1640 (18.2)	0.05
Missing # (%)	10 777 (22.5)	3938 (22.6)		6168 (20.0)		2263 (20.1)	
Dyslipidemia	12 607 (38.0)	4916 (39.8)	0.06	8595 (38.9)	0.06	3431 (41.3)	0.09
Missing # (%)	14 650 (30.6)	5073 (29.1)		8681 (28.2)		2942 (26.2)	
Cirrhosis	716 (2.0)	237 (1.9)	0.02	491 (2.1)	0.01	165 (1.9)	0.01
Missing # (%)	12 401 (25.9)	4618 (26.5)		7192 (23.4)		2658 (23.6)	
Neuropathy	2020 (5.7)	772 (6.0)	0.02	1485 (6.3)	0.08	565 (6.6)	0.05
Missing # (%)	12 233 (25.6)	4595 (26.4)		7067 (23.0)		2635 (23.4)	
Urological pathology	5111 (14.3)	2295 (17.9)	0.16	3488 (14.7)	0.03	1606 (18.6)	0.16
Missing # (%)	12 191 (25.5)	4594 (26.4)		7037 (22.9)		2632 (23.4)	
Smoking status			0.08		0.05		0.05
Non‐smoker	17 227 (53.1)	6596 (55.5)		11 364 (52.5)		4369 (54.5)	
Ex‐smoker	9160 (28.2)	3150 (26.5)		6097 (28.2)		2135 (26.7)	
Smoker	6044 (18.6)	2143 (18.0)		4170 (19.3)		1507 (18.8)	
Missing # (%)	15 411 (32.2)	5535 (31.8)		9151 (29.7)		3239 (28.8)	
CMV mismatch			0.10		0.11		0.03
*R+*	29 906 (73.1)	10 345 (70.5)		19 728 (74.8)		6885 (72.9)	
*D+ R−*	5237 (12.8)	1970 (13.4)		3185 (12.1)		1170 (12.4)	
*D− R−*	5776 (14.1)	2353 (16.0)		3474 (13.2)		1393 (14.7)	
Missing # (%)	6923 (14.5)	2759 (15.8)		4395 (14.3)		1802 (16.0)	
EBV mismatch			0.06		0.06		0.02
*R*+	45 127 (97.6)	16 258 (97.0)		29 212 (97.9)		10 588 (97.4)	
*D*+ *R−*	994 (2.2)	458 (2.7)		566 (1.9)		259 (2.4)	
*D− R−*	100 (0.2)	46 (0.3)		50 (0.2)		19 (0.2)	
Missing # (%)	1621 (3.4)	662 (3.8)		954 (3.1)		384 (3.4)	
# of kidney transplantation procedures per individual between 2005 and 2020			0.08		0.02		0.07
1	45 837 (95.8)	16 524 (94.8)		29 445 (95.7)		10 654 (94.7)	
2	1966 (4.1)	890 (5.1)		1313 (4.3)		591 (5.3)	
3	39 (0.1)	10 (0.1)		24 (0.1)		5 (0.0)	
Total # of transplant procedures	49 886	18 334		32 143		11 851	
Regional transplant activity # (%)							
Auvergne‐Rhone‐Alpes	5633 (13.5)	1344 (8.7)	1.09	4118 (13.7)	0.24	955 (8.6)	0.91
Bourgogne‐Franche‐Comté	1244 (3.0)	782 (5.1)		904 (3.0)		576 (5.2)	
Bretagne	1777 (4.3)	1166 (7.5)		1185 (3.9)		778 (7.0)	
Centre‐Val de Loire	1732 (4.2)	38 (0.2)		1200 (4.0)		27 (0.2)	
Départements et Régions d'outre‐mer	766 (1.8)	437 (2.8)		558 (1.9)		317 (2.9)	
Grand Est	3065 (7.4)	2446 (15.8)		2283 (7.6)		1831 (16.5)	
Hauts‐de‐France	2914 (7.0)	1425 (9.2)		2206 (7.3)		1048 (9.4)	
Ile‐de‐France	9372 (22.5)	2714 (17.5)		7334 (24.4)		2131 (19.2)	
Normandie	1904 (4.6)	1604 (10.4)		1383 (4.6)		1179 (10.6)	
Nouvelle‐Aquitaine	3603 (8.6)	1900 (12.3)		2255 (7.5)		1203 (10.8)	
Occitanie	4142 (9.9)	794 (5.1)		2697 (9.0)		504 (4.5)	
Pays de la Loire	3046 (7.3)	659 (4.3)		2193 (7.3)		452 (4.1)	
Provence‐Alpes‐Côte d'Azur	2485 (6.0)	170 (1.1)		1764 (5.9)		116 (1.0)	
Missing # (%)	8203 (16.4)	2855 (15.6)		2063 (6.4)		734 (6.2)	

*Note:* Percentages were calculated after exclusion of missing values. Information on missing data is provided only when applicable.

^a^ *n* (%) for categorical variables and median [q25–q75] for continuous variables.

^b^ Data are presented for the first transplantation, except for transplantation regions.

^c^ SMD: standardized mean difference.

The ISBA extract contains pharmacological data about immunosuppressive drugs, doses, exposure (*C*_min_, *C*_max_, AUC) and dose adjustment recommendations for at least one MISD, for 17 700 kidney grafts transplanted in 17 576 adult or pediatric patients between 2005 and 2020 (representing 36% of kidney grafts registered in CRISTAL); 15 676 of these patients (90%) were also identified in the SNDS database, and 11 250 in the final SNDS list (65%). Table S4 compares CRISTAL‐SNDS patients matched with ISBA or not, also showing generally small but sometimes statistically significant standardized mean differences (SMD), particularly regarding age at transplantation (less in patients matched with ISBA), as well as urological pathologies (more frequent in ISBA patients), and again a large difference regarding transplant center distribution. The completeness of the corresponding variables is described in Table S5, and the number of patients, transplants and drug exposure biomarkers available for analysis in Figure 2.

The type of data available in SNDS is vast. The variables are structured in 194 different tables of very varied size, most of which are renewed yearly (https://health‐data‐hub.shinyapps.io/dico‐snds/), for a total of 1660 tables. As an example, Table S6 shows the number of patients receiving each individual MISD at least once over their follow‐up and Table 6 shows the number of patients, transplants and AUCs in ISBA, sorted by patient age and MISD.

**TABLE 6:** Number of patients, transplants and AUCs in ISBA, sorted by patient age and maintenance immunosuppressive drug.

		Total	MMF	Tacrolimus	Cyclosporine	Everolimus	Sirolimus
Number of patients with at least 1 AUC	Adult	16 605	15 995	2381	1467	28	14
	Pediatric	832	782	413	112	0	0
	Unknown	242	227	34	62	0	1
	Total	17 424	16 769	2796	1629	28	15
Number of transplants with at least 1 AUC	Adult	16 852	16 230	2422	1469	28	14
	Pediatric	847	795	417	112	0	0
	Unknown	1	1	1	1	0	1
	Total	17 700	17 026	2840	1582	28	15
Number of AUCs	Adult	60 751	47 826	6576	6218	88	43
	Pediatric	7321	4633	2273	415	0	0
	Unknown	928	613	77	237	0	1
	Total	69 000	53 072	8926	6870	88	44

Abbreviation: MMF: mycophenolate mofetil.

## 4 Discussion

The PERP‐KT research program was made possible by the founding of the HDH in 2019, which provides secure storage for de‐identified healthcare data in full compliance with regulatory and ethical requirements. The HDH also offers a secure working environment for accessing and analyzing data (https://www.health‐data‐hub.fr).

PERP‐KT was selected in the first HDH call for projects, which funded the construction of the corresponding database. This database links kidney transplant patient data from three nationwide sources with complementary information—CRISTAL, ISBA, and SNDS—thereby creating an exceptional resource for pharmacoepidemiological studies in this field. However, obtaining all necessary regulatory and ethical approvals, securing data access, and performing data extraction, de‐identification, linkage, and cleaning required nearly 3 years before the first research projects could begin.

The demographic profile of the French kidney transplant population between 2005 and 2021, as extracted from the exhaustive CRISTAL registry, was broadly consistent with other registry‐based descriptions from other high‐income countries. Recipients were predominantly male (62.7%) and had a median age at first transplantation of 52 years. These figures are close to those reported by the ERA Registry for adult first kidney transplant recipients in Europe, where women represented 36% of recipients and the proportion of older recipients (≥ 65 years) increased over time, from 18% in 2010 to 28% in 2019 [5]. In contrast, large international differences were reported for primary renal disease. For the USA, the comparison with France is broadly consistent for age and sex: USRDS reports that the median age of US kidney transplant recipients in 2023 was 55 years, with over 60% men [6].

However, the main distinguishing feature of the French population was the relatively limited contribution of living donor transplantation, 12.4% over the study period, compared with approximately 23% in the USA [6] and substantially higher proportions in several European countries such as Nordic countries (27%–28%), Switzerland (34%) and the Netherlands (50%) [7]. In Korea, the transplant system relies much more heavily on living donation. In the KOTRY cohort, 2014–2019, 64.8% of kidney transplants were from living donors, the mean recipient age was 49.4 ± 11.5 years, and 59.7% of recipients were male [8].

Waiting time comparisons should be interpreted cautiously because registries differ in whether they report active waiting time, total waiting time, time from dialysis initiation, or time from listing. For instance, in the UK, NHSBT reported a median active waiting time of 502 days for adult deceased donor kidney‐only transplantation for registrations between April 2019 and March 2022, but a median total waiting time of 1169 days when dialysis and suspension periods were included [9].

Comparisons of smoking, CMV mismatch, EBV mismatch, and comorbidities were not possible because they are not uniformly reported in public registry annual reports, definitions may differ, and missingness in CRISTAL is around 22%–31%, which makes international benchmarking fragile.

Based on this French cohort, the PERP‐KT program encompasses four main research lines (see Supplemental Data for an overview), each subdivided into specific studies based on patient characteristics (e.g., pediatric, adult, elderly populations) and the immunosuppressive drugs considered. To date, four of these studies have received funding and three are expected to be published shortly.

The PERP‐KT project also has several limitations. First, episodes of graft rejection and biomarkers of drug exposure (e.g., trough concentrations of tacrolimus or cyclosporine) are not consistently collected by transplant centers within the Agence de la Biomédecine's CRISTAL registry. Fortunately, rejection episodes can still be identified in the SNDS whenever they result in hospital admission. In contrast, CRISTAL has been the only reliable source of graft loss. However, at the request of the HDH, work is underway to set up an algorithm to infer kidney graft loss from SNDS data. Also, the SNDS does not record treatments administered, imaging procedures or laboratory tests performed during hospital stays, which creates some uncertainty, for example, regarding therapies used to treat graft rejection. Nevertheless, MISDs prescribed at hospital discharge after transplantation are documented in CRISTAL. Finally, ISBA may not be the only model‐informed precision dosing (MIPD) tool used for MISDs in France, which could attenuate the apparent effect of ISBA when comparing outcomes in patients managed with versus without it, but in no way favor it.

Despite these challenges, and provided all adequate measures are taken to avoid biases as much as possible, the PERP‐KT database and research program are expected to deliver valuable real‐world evidence on: (i) the benefit–risk balance of MIPDs for maintenance immunosuppressants; (ii) the relationship between long‐term exposure and clinical effects, including identification of the most relevant biomarkers of drug exposure; and (iii) the most appropriate combinations and treatment sequences of MISDs according to patient profiles and medical history. In addition, the richness and longitudinal depth of clinical data in PERP‐KT provide a unique opportunity to model the trajectory of graft function decline over time, accounting for multiple, potentially competing risk factors. Such models may ultimately be used to predict and compare, in silico, the benefit–risk balance of different MISD strategies both at the population and individual levels.

## Author Contributions

**Pierre Marquet:** conception and design of the study, manuscript drafting and approval. **Sabrina Crépin:** data analysis and interpretation, manuscript drafting and approval. **Antoine Humeau and Clément Benoist:** data analysis and interpretation, manuscript drafting and approval. **Sylvain Couderc:** data analysis and interpretation, manuscript drafting and approval. **Caroline Monchaud:** design of the study, manuscript revision and approval. **Marc Labriffe:** design of the study, manuscript revision and approval. **Franck Saint‐Marcoux:** conception of the study, manuscript revision and approval.

## Funding

PERP‐KT has received funding and material support from the French Health Data Hub and the University Hospital of Limoges (CHU Limoges).

## Ethics Statement

The PERP‐KT program was approved by The French Committee on Informatics and Liberty (CNIL) on 20 November 2020 (authorization number 920315) and is registered by the Health Data Hub as T11068872019112. Due to the population size and time depth, CNIL approval includes the authorization of informing patients through their transplant physicians and the CHU Limoges and HDH websites, rather than individually.

## Conflicts of Interest

The authors declare no conflicts of interest.

## Supporting information


**Table S1:** ICD‐10, CCAM and GHM codes used for matching CRISTAL and SNDS patients, based on the « Méthodologie médicale de la cartographie des pathologies et des dépenses, version G9 (années 2015 à 2020, Tous Régimes), Caisse nationale d'assurance maladie, France ».
**Table S2:** Nature and completeness of CRISTAL variables.
**Table S3:** Characteristics at first transplantation of CRISTAL cases with confirmed or denied SNDS‐matching (2005–2020).
**Table S4:** Characteristics at first transplantation of SNDS‐matched CRISTAL cases according to ISBA matching status (2005–2020).
**Table S5:** Nature and completeness of ISBA variables.
**Figure S1:** Knowledge graph of the risks of kidney graft function decline over time.

## Data Availability

The data that support the findings of this study are available from Health Data Hub. Restrictions apply to the availability of these data, which were used under license for this study. Data are available from the author(s) with the permission of Health Data Hub.
