# Supplementary material for: A Pharmaco‐Epidemiological Research Program Leveraging a Nationwide Database of Kidney Transplantation
Source: Pharmacoepidemiol Drug Saf. 2026 Jul 23;35(8):e70442. doi: 10.1002/pds.70442 (PMC13396964; doi:10.1002/pds.70442)
Supplement: Supplementary file 1 — Table S1: ICD‐10, CCAM and GHM codes used for matching CRISTAL and SNDS patients, based on the « Méthodologie médicale de la cartographie des pathologies et des dépenses, version G9 (années 2015 à 2020, Tous Régimes), Caisse nationale d'assurance maladie, France ». Table S2: Nature and completeness of CRISTAL variables. Table S3: Characteristics at first transplantation of CRISTAL cases with confirmed or denied SNDS‐matching (2005–2020). Table S4: Characteristics at first transplantation of SNDS‐matched CRISTAL cases according to ISBA matching status (2005–2020). Table S5: Nature and completeness of ISBA variables. Figure S1: Knowledge graph of the risks of kidney graft function decline over time. [file PDS-35-e70442-s001.docx]

#### A Pharmaco-Epidemiological Research Program leveraging a Nationwide Database of Kidney Transplantation.

Pierre Marquet^1,2^, MD, PhD; Antoine Humeau^1^, PhD; Sabrina Crépin^1,2^, PharmD; Clément Benoist^2^, PhD; Sylvain Couderc^1,2^, PharmD; Caroline Monchaud^1,2^, PharmD, PhD; Marc Labriffe^1,2^, MD, PhD; Franck Saint-Marcoux^1,2^, PharmD, PhD.

^1^ Pharmacology & Transplantation, Université de Limoges, UMR1248 INSERM, Limoges, France.

^2^ Department of Pharmacology, Toxicology and Pharmacovigilance, CHU Limoges, Limoges, France.

**SUPPLEMENTARY MATERIAL**

1. **The main PERP-KT research lines**
   1. **Research line 1: Evaluation of model-informed precision dosing of the main maintenance immunosuppressive drugs (MISDs)**

### Context:

There is ample evidence that mycophenolate mofetil (MMF) is a narrow therapeutic index (NTI) drug, as stressed by the recent consensus report^1^. Since the predose plasma levels (C0) of mycophenolic acid (MPA), the active form of MMF, are not representative of overall MPA exposure or effects, due to highly variable entero-hepatic circulation, the drug is generally not monitored and dose adjusted, but rather prescribed at low doses to avoid adverse effects (AEs), resulting in poor efficacy or no efficacy at all (spurious treatment) in many patients^1^. Alternatively, we and other teams proposed to estimate MPA area under the concentration-time curve (AUC) in plasma, which requires dedicated tools (pharmacokinetics models and Bayesian estimators, or machine learning algorithms)^2–7^. A few clinical trials were performed to compare MMF AUC-based dose adjustment with standard dosing. In one of these, we found that it decreased the cumulative incidence of acute graft rejection episodes over the first year post-transplant^8^. All trials confirmed a strong AUC-efficacy relationship^9^. France may represent an exception regarding MMF, tacrolimus (TAC) and cyclosporine (CsA) dosing as many transplant centers use ISBA (ImmunoSuppressive Bayesian dose Adjustment expert system) to adjust drug dose based on the AUC since 2005. Each time an AUC is estimated on ISBA, Cmin and Cmax (minimum and maximum blood concentrations) are estimated as well. This real-world data represents a unique opportunity to address a pending question regarding these three drugs, employed in a vast majority of kidney transplant recipients: is AUC-based individual dose adjustment of these MISDs useful as compared to the standard of care, in terms of long-term efficacy and/or adverse events?

### Objectives

To compare treatment efficacy and AEs in patients “exposed” to MMF, TAC and/or CsA dose adjustment through the ISBA website to those not exposed. For each MISD, our primary objective is to evaluate, the clinical impact of using ISBA overall, and our secondary objectives to evaluate: (1) “preventive” dose adjustment, i.e. when the first AUC estimation is made early after transplantation, likely before any acute graft rejection; and (2) “reactive” dose adjustment, i.e. when the first AUC estimation is requested beyond the third month post-transplantation, or after the first acute graft rejection episode. The main outcome will be survival without graft loss of function. Secondary outcomes will be: the time to the occurrence of graft rejection, graft loss of function or patient death, as requested by the regulatory agencies for clinical trials in transplantation; survival without rejection; overall survival; as well as the cumulated incidence of severe adverse events (altogether as well as per category).

- 1. **Research line 2: Long term exposure – effects relationships of the main MISD**

### Context

Information about the long-time exposure – effect relationships of MISDs is lacking, and dosing recommendations mostly rely on 1-year up to 3-year comparative clinical trials, where only a few doses or exposure targets of a single drug were compared^10^. Our hypothesis is that knowing the best dosing strategies of maintenance ISDs for each type of patient profile, clinical condition, or even each patient would contribute to better use and harm-benefit balance of these drugs. Although drug doses are adjusted based on predose blood levels (C0) for calcineurin inhibitors and mTOR inhibitors, very little is known about a potential role of the peak concentration (Cmax) in efficacy or AEs. Also, C0 is used not because it is the biomarker best associated with efficacy or toxicity, but because it is practical and somewhat correlated with overall exposure (AUC, which is the mean concentration multiplied by the dosing interval: AUC = Caverage × interdose interval)^11–14^. For a given AUC, C0 and Cmax depend on the administration frequency as well as on the drug formulation. For instance, prolonged-release oral formulations of tacrolimus (TAC) allowing once daily instead of twice daily dosing have been approved. The AUC/C0 ratio of one of these is slightly higher than that of the historic formulation, which resulted in patient overdosing and overexposure (in terms of AUC) and an increased incidence of AEs at treatment inception^14^. For the other once-daily formulation, with an even slower release rate, researchers found decreased tremors when patients were switched from one of the immediate release formulations, which they hypothesized was due to lower Cmax^15^. Many pharmacokinetic (PK) studies were conducted and models developed to estimate tacrolimus inter-dose AUC and optimize dosing. However, there is no formal proof that it is superior to C0 for dose adjustment, in terms of harm-benefit balance. For another ISD, MMF, the situation is even more contrasted. It is administered twice daily, when corticosteroids and (increasingly) TAC are given once daily. Interestingly, tacrolimus and MPA have similar elimination half-lives, so that MMF might be given once daily, but to the best of our knowledge, there has been no study evaluating this. Whether it is feasible or not comes down to the relationships between MPA AUC, Cmax, Cmin and effects since giving MMF once daily in a double dose would result in higher Cmax and potentially lower Cmin, for theoretically identical AUC.

PERP-KT, where 36.4% of the kidney graft recipients in France over a 17-year period had MISDs AUC estimation made on ISBA (16,769, 2,796 and 1,629 patients with MPA, TAC or CsA AUCs, respectively) represents a unique opportunity to decipher the relationships between long term efficacy and AEs and the exposure biomarkers AUC, Cmin and Cmax.

### Objectives

The first objective is to evaluate statistical associations between MMF, TAC and CsA exposure biomarkers (Cmin, Cmax and AUC) taken individually and their evolution with time, and: death-censored graft survival (primary objective); patient survival; velocity of graft function decline (VGFD); AEs common to ISDs (severe infection, cancer); AEs more particularly attributable to MMF (severe digestive AEs, leucopenia, anemia, pancytopenia), TAC or CsA (major cardiovascular events, diabetes, neurological disorders).

The second objective is to evaluate statistical associations between combined exposure to MMF and TAC or MMF and CsA over time and: death-censored graft survival (primary objective); patient survival; velocity of graft function decline; AEs common to ISDs (infection, cancer); AEs attributable to MMF, TAC or CsA.

- 1. **Research line 3: MISDs treatment combinations & sequences for different patient profiles**

### Context

The current paradigm of evidence-based medicine does not readily apply to the increasing number of patients at the extremes of age, with comorbidities^17^ or polypharmacy, because they are frequently excluded from clinical trials. This is particularly the case in kidney transplantation, where a steadily increasing percentage of donors and recipients are very young or elderly, with a high impact on death-censored graft survival. The patients who participated in the registration trials of the MISD, or even in later randomized controlled trials that influenced maintenance treatments worldwide^10,18^, were generally of middle age, with few comorbidities and not polymedicated. Also, no second-line treatment strategy has been clinically validated after serious adverse events such as post-transplantation proliferative disorders (PTLD), solid cancer (with the notable exception of skin cancer^19,20^) or serious infectious diseases. One possible way to improve IS treatment harm-benefit balance in kidney transplantation is to identify the best MISDs combinations at the extremes of age, and the best second-line combinations after serious comorbidities, for the most frequent patient profiles, or even for each patient. This is obviously beyond the reach of randomized clinical trials, but it typically falls in the realm of precision medicine. Finely time-adjusted sequences of ISD combinations inferred from real-world data may outperform the standard-of-care ISD regimes in patients with risk profiles at either end of the spectrum, i.e. patients at very low or very high risk of rejection, as well as in patients with pre-existing or emerging comorbidities modifying patient or graft life-expectancy (such as cancer or chronic infection). In the same vein although not (yet) at the individual level, a reinforcement learning model has been developed to aid with the selection of optimal drugs for first- and second-line treatments and of the optimal time to initiate second line when designing non-small cell lung cancer trials^21^. AI now opens capabilities to derive insights about treatment effects from real-world data, which can be accounted for in patient care guidelines, whether such guidelines were previously based on randomized control trials (generally conducted in a selected population and not available for every situation) or on expert opinions (with a rather low level of evidence).

### Objectives

The main objective is to generate evidence from real-world data supporting updated recommendations about MISDs combinations and sequences in kidney transplantation, in the main clinical situations overlooked by, and over a time span beyond the reach of, randomized controlled clinical trials. These include patients at extremes of age, or after serious comorbidities, or on the contrary with apparently excellent tolerance towards the graft.

The long-term efficacy of the most frequent maintenance treatment strategies in kidney transplant recipients will be evaluated: in the overall cohort; in patients who showed no sign of graft rejection at 10 years after transplantation; in patients aged less than 5 or 10 years, or more than 70 years or 80 years at transplantation; after a diagnosis of cancer (of the skin, or solid cancer); after a diagnosis of PTLD; after a diagnosis of severe chronic viral infection (cytomegalovirus, BK virus), or tuberculosis, or fungal infection.

- 1. **Research line 4: Modelling the velocity of graft function deterioration to predict treatment effects**

### Context

Several research groups have developed statistical tools or models to predict kidney graft survival, or failure rate, at a time horizon of 1 to 10 years, based on graft function and a few risk factors at one-year post-transplantation^22,23^. However, none of these tools account for the nature of the initial kidney disease, patient comorbidities or drug nephrotoxicity, for instance, all of which may precipitate graft function decline and ultimately graft loss. Also, none of these models can simulate the evolution of graft function depending on different MISDs regimens, which would help transplant physicians make therapeutic decisions. AI alone or combined with pathophysiological models (knowledge graphs) now offers the possibility to grasp all these potential risk factors^24^ and to re-evaluate patient and graft life expectancy after adverse clinical events or treatment modification, as well as to compare virtually different treatment options at each step of patient care, for instance for rare populations excluded from registration clinical trials (infants and children, or the elderly).

The authors developed and filed for protection by Inserm-Transfer, a knowledge graph^25^ entitled “risk network in transplantation”, in which the primary risks of graft dysfunction are favored by secondary and possibly tertiary risks (supplemental Figure 1). This knowledge graph comes with the following hypotheses: (i) the different types of risks may cause harm at different times post-transplantation and/or with different frequencies depending on patient profiles; (ii) at a given post-transplant period in an individual, only a few of these potential risks harm the graft; (iii) biomarkers can be used as early surrogates of the harm being caused by certain risks; (iv) the different risks and biomarkers can be combined into a conditional network; and (v) MISDs as well as other drugs have preventive effects on certain risks (e.g., of graft rejection, graft vasculopathy, major cardiovascular events, etc.), but may also have AEs additive to or synergistic with certain risks (e.g., nephrotoxicity, metabolic AEs, hypertension, vasculopathy, etc.).

### Objectives

A posteriori, longitudinal evaluation of the clinical, laboratory and pharmacological variables associated with VGFD will be leveraged to develop a hybrid, dynamic (time-dependent), deep learning model able to forecast VGFD at time horizons of 2, 5 and 10 years, with uncertainty computation, as a platform to evaluate and compare the harm-benefit balance of different MISDs regimens at the individual level.

**1.5 References**

1. Bergan S, Brunet M, Hesselink DA, et al. Personalized Therapy for Mycophenolate: Consensus Report by the International Association of Therapeutic Drug Monitoring and Clinical Toxicology. *Ther Drug Monit*. 2021;43(2):150-200. doi:10.1097/FTD.0000000000000871

2. Prémaud A, Le Meur Y, Debord J, et al. Maximum a posteriori bayesian estimation of mycophenolic acid pharmacokinetics in renal transplant recipients at different postgrafting periods. *Ther Drug Monit*. 2005;27(3):354-361. doi:10.1097/01.ftd.0000162231.90811.38

3. Prémaud A, Debord J, Rousseau A, et al. A double absorption-phase model adequately describes mycophenolic acid plasma profiles in de novo renal transplant recipients given oral mycophenolate mofetil. *Clin Pharmacokinet*. 2005;44(8):837-847. doi:10.2165/00003088-200544080-00005

4. Prémaud A, Weber LT, Tönshoff B, et al. Population pharmacokinetics of mycophenolic acid in pediatric renal transplant patients using parametric and nonparametric approaches. *Pharmacol Res*. 2011;63(3):216-224. doi:10.1016/j.phrs.2010.10.017

5. Monchaud C, De Winter B, Premaud A, et al. Bayesian estimation of mycophenolate mofetil (MMF) in lung transplantation using a population pharmacokinetic model developed in renal and lung transplant recipients. *Fundam Clin Pharmacol*. 2011;25:46-46.

6. de Winter BCM, Monchaud C, Prémaud A, et al. Bayesian estimation of mycophenolate mofetil in lung transplantation, using a population pharmacokinetic model developed in kidney and lung transplant recipients. *Clin Pharmacokinet*. 2012;51(1):29-39. doi:10.2165/11594050-000000000-00000

7. Woillard JB, Labriffe M, Debord J, Marquet P. Mycophenolic acid exposure prediction using machine learning. *Clin Pharmacol Ther*. Published online February 24, 2021. doi:10.1002/cpt.2216

8. Le Meur Y, Büchler M, Thierry A, et al. Individualized mycophenolate mofetil dosing based on drug exposure significantly improves patient outcomes after renal transplantation. *Am J Transplant Off J Am Soc Transplant Am Soc Transpl Surg*. 2007;7(11):2496-2503. doi:10.1111/j.1600-6143.2007.01983.x

9. Metz DK, Holford N, Kausman JY, et al. Optimizing Mycophenolic Acid Exposure in Kidney Transplant Recipients: Time for Target Concentration Intervention. *Transplantation*. 2019;103(10):2012-2030. doi:10.1097/TP.0000000000002762

10. Ekberg H, Tedesco-Silva H, Demirbas A, et al. Reduced Exposure to Calcineurin Inhibitors in Renal Transplantation. *N Engl J Med*. 2007;357(25):2562-2575. doi:10.1056/NEJMoa067411

11. Haverals L, Roosens L, Wouters K, et al. Does the Tacrolimus Trough Level Adequately Predict Drug Exposure in Patients Requiring a High Tacrolimus Dose? *Transplant Direct*. 2023;9(4):e1439. doi:10.1097/TXD.0000000000001439

12. Marquet P, Cros F, Micallef L, et al. Tacrolimus Bayesian Dose Adjustment in Pediatric Renal Transplant Recipients. *Ther Drug Monit*. 2021;43(4):472-480. doi:10.1097/FTD.0000000000000828

13. Marquet P, Albano L, Woillard JB, et al. Comparative clinical trial of the variability factors of the exposure indices used for the drug monitoring of two tacrolimus formulations in kidney transplant recipients. *Pharmacol Res*. 2018;129:84-94. doi:10.1016/j.phrs.2017.12.005

14. Caillard S, Moulin B, Buron F, et al. Advagraf ^®^ , a once-daily prolonged release tacrolimus formulation, in kidney transplantation: literature review and guidelines from a panel of experts. *Transpl Int*. 2016;29(8):860-869. doi:10.1111/tri.12674

15. Langone A, Steinberg SM, Gedaly R, et al. Switching STudy of Kidney TRansplant PAtients with Tremor to LCP-TacrO (STRATO): an open-label, multicenter, prospective phase 3b study. *Clin Transplant*. 2015;29(9):796-805. doi:10.1111/ctr.12581

17. Liu F, Panagiotakos D. Real-world data: a brief review of the methods, applications, challenges and opportunities. *BMC Med Res Methodol*. 2022;22(1):287. doi:10.1186/s12874-022-01768-6

18. De Simone P, Nevens F, De Carlis L, et al. Everolimus with reduced tacrolimus improves renal function in de novo liver transplant recipients: a randomized controlled trial. *Am J Transplant Off J Am Soc Transplant Am Soc Transpl Surg*. 2012;12(11):3008-3020. doi:10.1111/j.1600-6143.2012.04212.x

19. Dantal J, Morelon E, Rostaing L, et al. Sirolimus for Secondary Prevention of Skin Cancer in Kidney Transplant Recipients: 5-Year Results. *J Clin Oncol Off J Am Soc Clin Oncol*. 2018;36(25):2612-2620. doi:10.1200/JCO.2017.76.6691

20. Euvrard S, Morelon E, Rostaing L, et al. Sirolimus and secondary skin-cancer prevention in kidney transplantation. *N Engl J Med*. 2012;367(4):329-339. doi:10.1056/NEJMoa1204166

21. Zhao Y, Zeng D, Socinski MA, Kosorok MR. Reinforcement learning strategies for clinical trials in nonsmall cell lung cancer. *Biometrics*. 2011;67(4):1422-1433. doi:10.1111/j.1541-0420.2011.01572.x

22. Prémaud A, Filloux M, Gatault P, et al. An adjustable predictive score of graft survival in kidney transplant patients and the levels of risk linked to de novo donor-specific anti-HLA antibodies. Stepkowski S, ed. *PLOS ONE*. 2017;12(7):e0180236. doi:10.1371/journal.pone.0180236

23. Stamenic D, Rousseau A, Essig M, et al. A Prognostic Tool for Individualized Prediction of Graft Failure Risk within Ten Years after Kidney Transplantation. *J Transplant*. 2019;2019:7245142. doi:10.1155/2019/7245142

24. Lee BK, Lessler J, Stuart EA. Improving propensity score weighting using machine learning. *Stat Med*. 2010;29(3):337-346. doi:10.1002/sim.3782

25. MacLean F. Knowledge graphs and their applications in drug discovery. *Expert Opin Drug Discov*. 2021;16(9):1057-1069. doi:10.1080/17460441.2021.1910673

1. **Supplemental tables**

**Supplemental Table 1: ICD-10, CCAM and GHM codes used for matching CRISTAL and SNDS patients, based on the « Méthodologie médicale de la cartographie des pathologies et des dépenses, version G9 (années 2015 à 2020, Tous Régimes), Caisse nationale d’assurance maladie, France ».**

| **Coding source** | **Disease** | **Code** |
| --- | --- | --- |
| GHM^a^ | Kidney transplantation | 27C06Z, 27C061, 27C062, 27C063, 27C064 |
|  | Kidney transplantation follow-up | 24M39Z, 11M171, 11M172, 11M173, 11M174 |
|  | Renal insufficiency with dialysis | 11K021, 11K022, 11K023, 11K024, 11K02J |
|  | Peritoneal dialysis or haemodialysis | 28Z01Z, 28Z02Z, 28Z03Z, 28Z04Z, 28Z05Z, 28Z06Z |
| CCAM^b^ | Pancreas and kidney transplantation | HNEA002 |
|  | Kidney transplantation | JAEA003 |
|  | Kidney biopsy | JAHB001, JAHH002, JAHJ006, JAHJ007, JAHC001, ZZHH008 |
|  | Dialysis | JVJB001, JVJF004, JVJF008, JVRP004, JVRP007, JVRP008, YYYY007 |
|  | Doppler ultrasound of the kidney and its vessels | JAQM002 |
| ICD-10 | End-stage renal disease | N180, N185 |
|  | Kidney transplantation | Z940 |
|  | Dialysis | Z491 |

^a^ Diagnosis-Related Groups (DRGs) used in the French hospital information system, referred to as ‘Groupes Homogènes de Malades’ (GHM), classify hospital stays into clinically and economically homogeneous categories based on medical diagnoses, procedures, age, and other factors. They are primarily used for hospital financing and activity reporting

^b^ The French Common Classification of Medical Procedures (‘Classification Commune des Actes Médicaux’, CCAM) is a standardized coding system used in France to describe diagnostic and therapeutic medical procedures for hospital and outpatient care.

**Supplemental Table 2. Nature and completeness of CRISTAL variables**

| **Variables** | **Missing data (%)** |
| --- | --- |
| ***At or prior to the time of transplantation (N = 49,886 transplants)*** | |
| Donor age at tx*** | 8 (0) |
| Recipient age at tx | 0 |
| Donor sex | 8 (0) |
| Recipient sex | 0 |
| Donor vital status | 1 (0) |
| Transplant rank (inside the PERP-KT cohort) | 0 |
| Donor height | 101 (0) |
| Recipient height | 2,554 (5) |
| Donor weight | 103 (0) |
| Recipient weight at registration | 1,966 (4) |
| Recipient weight at hospital discharge | 8,215 (16) |
| Cold ischemia time | 1,614 (3) |
| Warm ischemia time | 20,193 (40) |
| Crossmatch | 1,835 (4) |
| Hyperimmunization | 17,902 (36) |
| HLA-A incompatibilities | 72 (0) |
| HLA-B incompatibilities | 72 (0) |
| HLA-DR incompatibilities | 72 (0) |
| HLA-DQ incompatibilities | 31,049 (62) |
| Incompatibility graft rate | 17,310 (35) |
| Immunosuppressive induction treatment | 39,321 (79) |
| Immunoglobulines between -30 days and tx | 21,084 (42) |
| Immunoglobulines after tx | 39,046 (78) |
| Plasmapheresis between -30 days and tx | 20,985 (42) |
| Plasmapheresis after tx | 39,093 (78) |
| Immunoadsorption between -30 days and tx | 39,239 (79) |
| Immunoadsorption after tx | 39,101 (78) |
| Rituximab between -30 days and tx | 20,909 (42) |
| Rituximab after tx | 39,096 (78) |
| Bortezomib between -30 days and tx | 21,238 (43) |
| Bortezomib after tx | 39,102 (78) |
| Eculizumab between -30 days and tx | 21,224 (43) |
| Eculizumab after tx | 39,099 (78) |
| Tocilizumab between -30 days and tx | 39,240 (79) |
| Tocilizumab after tx | 39,102 (78) |
| Carfilzomib between -30 days and tx | 39,240 (79) |
| Carfilzomib after tx | 39,102 (78) |
| Maintenance immunosuppressive treatment |  |
| Corticosteroids | 785 (2) |
| Calcineurin inhibitors | 38,661 (77) |
| Antimetabolites | 39,051 (78) |
| mTOR inhibitors | 49,377 (99) |
| Belatacept | 38,566 (77) |
| ***Annual follow-up post-transplantation*** | ***N = 344,804*** |
| Serum creatinine | 10,587 (3) |
| DSA de novo | 234,221 (68) |
| Daily activity | 136,022 (39) |
| Height | 189,404 (55) |
| Weight | 72,975 (21) |
| BKV status | 341,930 (99) |
| CMV status | 341,495 (99) |
| EBV status | 341,299 (99) |
| Proteinuria in g/24h | 237,389 (69) |
| Proteinuria in g/L | 190,447 (55) |

**tx: transplantation*

**Supplemental Table 3. Characteristics at first transplantation of CRISTAL cases with confirmed or denied SNDS-matching (2005 – 2020)**

| **Patient characteristics^a^** | **Confirmed SNDS matching**  **N=30,782** | **Denied SNDS matching**  **N=11,923** | **SMD^c^** | **p-value^b^** |  |
| --- | --- | --- | --- | --- | --- |
| **Donor vital status** (living donor) | 3,248 (10.6) | 1,902 (16.0) | 0.16 | **<0.01** |  |
| **Time spent on waiting list** (y) | 1. [1.0-3.0] | 1. [1.0-2.0] | 0.12 | **<0.01** |  |
| *Missing # (%)* | *2 (0.0)* | *2 (0.0)* |  |  |  |
| **Age at registration on the waiting list registration** (y) | 50.0 [39.0-60.0] | 51.0 [37.0-61.0] | 0.02 | 0.76 |  |
| **Age at first transplant** (y) | 53.0 [41.0-62.0] | 53.0 [39.0-63.0] | 0.04 | 0.45 |  |
| **Age categories** (y): |  |  | 0.18 | **<0.01** |  |
| [0-10[ | 350 (1.1) | 125 (1.0) |  |  |  |
| [10-20[ | 612 (2.0) | 402 (3.4) |  |  |  |
| [20-30[ | 1,793 (5.8) | 1,078 (9.0) |  |  |  |
| [30-40[ | 3,962 (12.9) | 1,415 (11.9) |  |  |  |
| [40-50[ | 6,258 (20.3) | 1,959 (16.4) |  |  |  |
| [50-60[ | 7,892 (25.6) | 2,912 (24.4) |  |  |  |
| [60-70[ | 7,030 (22.8) | 2,768 (23.2) |  |  |  |
| [70-80[ | 2,728 (8.9) | 1,189 (10.0) |  |  |  |
| [80-90[ | 157 (0.5) | 75 (0.6) |  |  |  |
| ***Sex*** *(male)* | 18,808 (61.1) | 8,074 (67.7) | 0.14 | **<0.01** |  |
| **BMI at registration on the waiting list** (kg/m²) | 24.3 [21.5-27.8] | 24.2 [21.4-27.5] | 0.03 | **0.02** |  |
| *Missing # (%)* | *1,551 (5.0)* | *570 (4.8)* |  |  |  |
| **Comorbidities at registration on the waiting list** |  |  |  |  |  |
| Arrhythmia | 1,036 (4.4) | 458 (4.8) | 0.02 | 0.11 |  |
| *Missing # (%)* | *7,130 (23.2)* | *2,361 (19.8)* |  |  |  |
| Coronary insufficiency | 1,808 (7.7) | 689 (7.2) | 0.02 | 0.17 |  |
| *Missing # (%)* | *7,216 (23.4)* | *2,390 (20.0)* |  |  |  |
| Heart failure | 1,007 (4.3) | 384 (4.0) | 0.01 | 0.3 |  |
| *Missing # (%)* | *7,356 (23.9)* | *2,411 (20.2)* |  |  |  |
| Stroke (ischemic/haemorrhagic) | 913 (3.8) | 334 (3.5) | 0.02 | 0.12 |  |
| *Missing # (%)* | *6,968 (22.6)* | *2,297 (19.3)* |  |  |  |
| Myocardial infarction | 1,037 (4.4) | 409 (4.3) | 0.01 | 0.68 |  |
| *Missing # (%)* | *7,029 (22.8)* | *2,313 (19.4)* |  |  |  |
| Unstable angina pectoris | 246 (1.0) | 96 (1.0) | <0.01 | 0.81 |  |
| *Missing # (%)* | *7,124 (23.1)* | *2,348 (19.7)* |  |  |  |
| Transient ischemic attack | 534 (2.3) | 216 (2.3) | <0.01 | 1.00 |  |
| *Missing # (%)* | *7,079 (23.0)* | *2,338 (19.6)* |  |  |  |
| Hypertension | 16,221 (69.3) | 6,411 (67.6) | 0.04 | **<0.01** |  |
| *Missing # (%)* | *7,361 (23.9)* | *2,439 (20.5)* |  |  |  |
| Diabetes | 5,132 (20.8) | 1,694 (17.1) | 0.09 | **<0.01** |  |
| *Missing # (%)* | *6,168 (20.0)* | *2,044 (17.1)* |  |  |  |
| Dyslipidemia | 8,595 (38.9) | 3,333 (37.1) | 0.04 | **<0.01** |  |
| *Missing # (%)* | *8,681 (28.2)* | *2,935 (24.6)* |  |  |  |
| Cirrhosis | 491 (2.1) | 183 (1.9) | 0.01 | 0.36 |  |
| *Missing # (%)* | *7,192 (23.4)* | *2,385 (20.0)* |  |  |  |
| Neuropathy | 1,485 (6.3) | 446 (4.7) | 0.07 | **<0.01** |  |
| *Missing # (%)* | *7,067 (23.0)* | *2,337 (19.6)* |  |  |  |
| Urological pathology | 3,488 (14.7) | 1,391 (14.5) | 0.01 | 0.68 |  |
| *Missing # (%)* | *7,037 (22.9)* | *2,332 (19.6)* |  |  |  |
| Smoking status |  |  | 0.05 | **<0.01** |  |
| *Non-smoker* | 11,364 (52.5) | 4,644 (53.4) |  |  |  |
| *Ex-smoker* | 6,097 (28.2) | 2,552 (29.4) |  |  |  |
| *Smoker* | 4,170 (19.3) | 1,499 (17.2) |  |  |  |
| *Missing # (%)* | *9,151 (29.7)* | *3,228 (27.1)* |  |  |  |
| CMV mismatch |  |  | 0.20 | **<0.01** |  |
| *R+* | 19,728 (74.8) | 6,484 (65.9) |  |  |  |
| *D+R-* | 3,185 (12.1) | 1,561 (15.9) |  |  |  |
| *D-R-* | 3,474 (13.2) | 1,797 (18.3) |  |  |  |
| *Missing # (%)* | *4,395 (14.3)* | *2,081 (17.5)* |  |  |  |
| EBV mismatch |  |  | 0.05 | **<0.01** |  |
| *R+* | 29,212 (97.9) | 11,154 (97.1) |  |  |  |
| *D+R-* | 566 (1.9) | 307 (2.7) |  |  |  |
| *D-R-* | 50 (0.2) | 28 (0.2) |  |  |  |
| *Missing # (%)* | *954 (3.1)* | *434 (3.6)* |  |  |  |
| **Number of kidney transplantation procedures per individual, 2005 to 2020** | | |  |  |  |
| *1 n (%)* | 29,445 (95.7) | 11,345 (95.2) | 0.03 | 0.06 |  |
| *2 n (%)* | 1,313 (4.3) | 565 (4.7) |  |  |  |
| *3 n (%)* | 24 (0.1) | 13 (0.1) |  |  |  |
| Total *# of transplant procedures* | 32,143 | 12,514 |  |  |  |

Notes:

^a^ n (%) for categorical variables and median [q25-q75] for continuous variables

^b^ Differences between groups were assessed using the χ² test for categorical variables and the Mann‑Whitney test for continuous variables.

^c^ SMD: standardized mean difference

Percentages were calculated after exclusion of missing values. Information on missing data is provided only when applicable.

.

**Supplemental Table 4. Characteristics at first transplantation of SNDS-matched CRISTAL cases according to ISBA matching status (2005–2020)**

| **Patient characteristics^a^** | **CRISTAL-SNDS matched with ISBA**  **N=11,250** | | **CRISTAL-SNDS not matched with ISBA**  **N=19,532** | | **SMD** | **p-value^b^** |  |
| --- | --- | --- | --- | --- | --- | --- | --- |
| **Donor vital status** (living donor) | 1,094 (9.7) | | 2,154 (11.0) | | 0.04 | <0.01 |  |
| **Time spent on waiting list** (y) | 1.0 [1.0-3.0] | | 1.0 [1.0-3.0] | | 0.08 | <0.01 |  |
| **Age at registration on the waiting list registration** (y) | 49.0 [38.0-59.0] | | 51.0 [40.0-61.0] | | 0.17 | <0.01 |  |
| **Age at first transplant** (y) | 51.0 [40.0-61.0] | | 53.0 [42.0-63.0] | | 0.18 | <0.01 |  |
| **Age categories** (y): |  | |  | |  |  |  |
| [0-10[ | 182 (1.6) | | 168 (0.9) | | 0.20 | <0.01 |  |
| [10-20[ | 332 (3.0) | | 280 (1.4) | |  |  |  |
| [20-30[ | 724 (6.4) | | 1,069 (5.5) | |  |  |  |
| [30-40[ | 1,536 (13.7) | | 2,426 (12.4) | |  |  |  |
| [40-50[ | 2,406 (21.4) | | 3,852 (19.7) | |  |  |  |
| [50-60[ | 2,869 (25.5) | | 5,023 (25.7) | |  |  |  |
| [60-70[ | 2,424 (21.5) | | 4,,606 (23.6) | |  |  |  |
| [70-80[ | 743 (6.6) | | 1985 (10.2) | |  |  |  |
| [80-90[ | 34 (0.3) | | 123 (0.6) | |  |  |  |
| ***Sex*** *(male)* | 6,833 (60.7) | | 11,975 (61.3) | | 0.01 | 0.33 |  |
| **BMI at registration on the waiting list** (kg/m²) | 24.2 [21.2-27.8] | | 24.4 [21.5-27.8] | | 0.04 | <0.01 |  |
| *Missing # (%)* | *554 (4.9)* | | *997 (5.1)* | |  |  |  |
| **Comorbidities at registration on the waiting list** |  | |  | |  |  |  |
| Arrhythmia | 359 (4.2) | | 677 (4.5) | | 0.02 | 0.26 |  |
| *Missing # (%)* | *2,655 (23.6)* | | *4,475 (22.9)* | |  |  |  |
| Coronary insufficiency | 607 (7.1) | | 1201 (8.0) | | 0.04 | 0.01 |  |
| *Missing # (%)* | *2,679 (23.8)* | | *4,537 (23.2)* | |  |  |  |
| Heart failure | 336 (3.9) | | 671 (4.5) | | 0.03 | 0.04 |  |
| *Missing # (%)* | *2,689 (23.9)* | | *4,667 (23.9)* | |  |  |  |
| Stroke (ischemic/haemorrhagic) | *300 (3.5)* | | *613 (4.0)* | | 0.03 | 0.03 |  |
| *Missing # (%)* | *2,620 (23.3)* | | *4,348 (22.3)* | |  |  |  |
| Myocardial infarction | *346 (4.0)* | | *691 (4.6)* | | 0.03 | 0.05 |  |
| *Missing # (%)* | *2,628 (23.4)* | | *4,401 (22.5)* | |  |  |  |
| Unstable angina pectoris | *86 (1.0)* | | *160 (1.1)* | | 0.01 | 0.69 |  |
| *Missing # (%)* | *2,646 (23.5)* | | *4,478 (22.9)* | |  |  |  |
| Transient ischemic attack | *212 (2.5)* | | *322 (2.1)* | | 0.02 | 0.11 |  |
| *Missing # (%)* | *2,634 (23.4)* | | *4445 (22.8)* | |  |  |  |
| Hypertension | *6,160 (71.9)* | | *10,067 (67.8)* | | 0.09 | <0.01 |  |
| *Missing # (%)* | *2,677 (23.8)* | | *4,684 (24.0)* | |  |  |  |
| Diabetes | *1,640 (18.2)* | | *3,492 (22.3)* | | 0.10 | <0.01 |  |
| *Missing # (%)* | *2,263 (20.1)* | | *3,905 (20.0)* | |  |  |  |
| Dyslipidemia | *3,431 (41.3)* | | *5,164 (37.4)* | | 0.08 | <0.01 |  |
| *Missing # (%)* | *2,942 (26.2)* | | *5,739 (29.4)* | |  |  |  |
| Cirrhosis | *165 (1.9)* | | *326 (2.2)* | | 0.02 | 0.21 |  |
| *Missing # (%)* | *2,658 (23.6)* | | *4,534 (23.2)* | |  |  |  |
| Neuropathy | *656 (6.6)* | | *920 (6.1)* | | 0.02 | 0.16 |  |
| *Missing # (%)* | *2,635 (23.4)* | | *4,432 (22.7)* | |  |  |  |
| Urological pathology | *1,606 (18.6)* | | *1,882 (12.4)* | | 0.17 | <0.01 |  |
| *Missing # (%)* | *2,632 (23.4)* | | *4,405 (22.6)* | |  |  |  |
| Smoking status |  | |  | |  |  |  |
| *Non-smoker* | 4,369 (54.5) | | 6,995 (51.4) | | 0.07 | <0.01 |  |
| *Ex-smoker* | 2,135 (26.7) | | 3,962 (29.1) | |  |  |  |
| *Smoker* | 1,507 (18.8) | | 2,663 (19.6) | |  |  |  |
| *Missing # (%)* | *3,239 (28.8)* | | *5,912 (30.3)* | |  |  |  |
| CMV mismatch |  | |  | |  |  |  |
| *R+* | 6,885 (72.9) | | 12,843 (75.8) | |  |  |  |
| *D+R-* | 1,170 (12.4) | | 2,015 (11.9) | |  |  |  |
| *D-R-* | 1,393 (14.7) | | 2,081 (12.3) | | 0.08 | <0.01 |  |
| *Missing # (%)* | *1,802 (16.0)* | | *2,593 (13.3)* | |  |  |  |
| EBV mismatch |  | |  | |  |  |  |
| *R+* | 10,588 (97.4) | | 18,624 (98.2) | | 0.05 | <0.01 |  |
| *D+R-* | 259 (2.4) | | 307 (1.6) | |  |  |  |
| *D-R-* | 19 (0.2) | | 31 (0.2) | |  |  |  |
| *Missing # (%)* | *384 (3.4)* | | *570 (2.9)* | |  |  |  |
| **Number of kidney transplantation procedures per individual, 2005 to 2020** | | | | |  |  |  |
| *1 n (%)* | | 10,654 (94.7) | | 18,791 (96.2) | 0.08 | <0.01 |  |
| *2 n (%)* | | 591 (5.3) | | 722 (3.4) |  |  |  |
| *3 n (%)* | | 5 (0.0) | | 19 (0.1) |  |  |  |
| Total *# of transplant procedures* | | 11,851 | | 20,292 |  |  |  |
| **Regional transplant activity # (%)** | |  | |  | 1.10 | <0.01 |  |
| Auvergne-Rhone-Alpes | | 955 (8.6) | | 3,163 (16.7) |  |  |  |
| Bourgogne-Franche-Comté | | 576 (5.2) | | 328 (1.7) |  |  |  |
| Bretagne | | 778 (7.0) | | 407 (2.1) |  |  |  |
| Centre-Val de Loire | | 27 (0.2) | | 1,173 (6.2) |  |  |  |
| Départements et Régions d’outre-mer | | 317 (2.9) | | 241 (1.3) |  |  |  |
| Grand Est | | 1,831 (16.5) | | 452 (2.4) |  |  |  |
| Hauts-de-France | | 1,048 (9.4) | | 1,158 (6.1) |  |  |  |
| Ile-de-France | | 2,131 (19.2) | | 5,203 (27.4) |  |  |  |
| Normandie | | 1,179 (10.6) | | 204 (1.1) |  |  |  |
| Nouvelle-Aquitaine | | 1,203 (10.8) | | 1,052 (5.5) |  |  |  |
| Occitanie | | 504 (4.5) | | 2,193 (11.6) |  |  |  |
| Pays de la Loire | | 452 (4.1) | | 1,741 (9.2) |  |  |  |
| Provence-Alpes-Côte d’Azur | | 116 (1.0) | | 1,648 (8.7) |  |  |  |
| *Missing # (%)* | | *734 (6.2)* | | *1,329 (6.5)* |  |  |  |

Notes:

^a^ n (%) for categorical variables and median [q25-q75] for continuous variables

^b^ Differences between groups were assessed using the χ² test for categorical variables and the Mann‑Whitney test for continuous variables.

Percentages were calculated after exclusion of missing values. Information on missing data is provided only when applicable.

**Supplemental Table 5. Nature and completeness of ISBA variables**

| **Variables** | **Missing data (%)** |
| --- | --- |
| Patient age | 16 (0) |
| Transplantation date | *928 (1)* |
| MISDs for which dose adjustment was requested |  |
| *Nature of IS drug* | 0 (0) |
| *Daily Dose* | 4 (0) |
| *Precise time of last dosing* | 10 (0) |
| *Time between transplant and blood sampling* | 21 (0) |
| *At least three whole blood or plasma concentrations, with sampling times* | 16,425 (24) |
| *Measured Cmin*  *(or calculated Cmin for mycophenolate)* | 221 (0) |
| *Calculated Cmax* | 2 (0) |
| *Calculated AUC* | 1 (0) |
| Cause of AUC requests | 20,086 (29) |

**Supplemental Table 6. MISDs usage based on the SNDS**

| **# patients on each MISD (%)** | **N** | **(%)** |
| --- | --- | --- |
| Mycophenolate | 28,555 | (93) |
| Tacrolimus | 25,370 | (82) |
| Azathioprine | 3,711 | (12) |
| Cyclosporine | 7,411 | (24) |
| Sirolimus | 1,354 | (4) |
| Everolimus | 4,523 | (15) |
|  |  |  |
|  |  |  |
|  |  |  |

1.
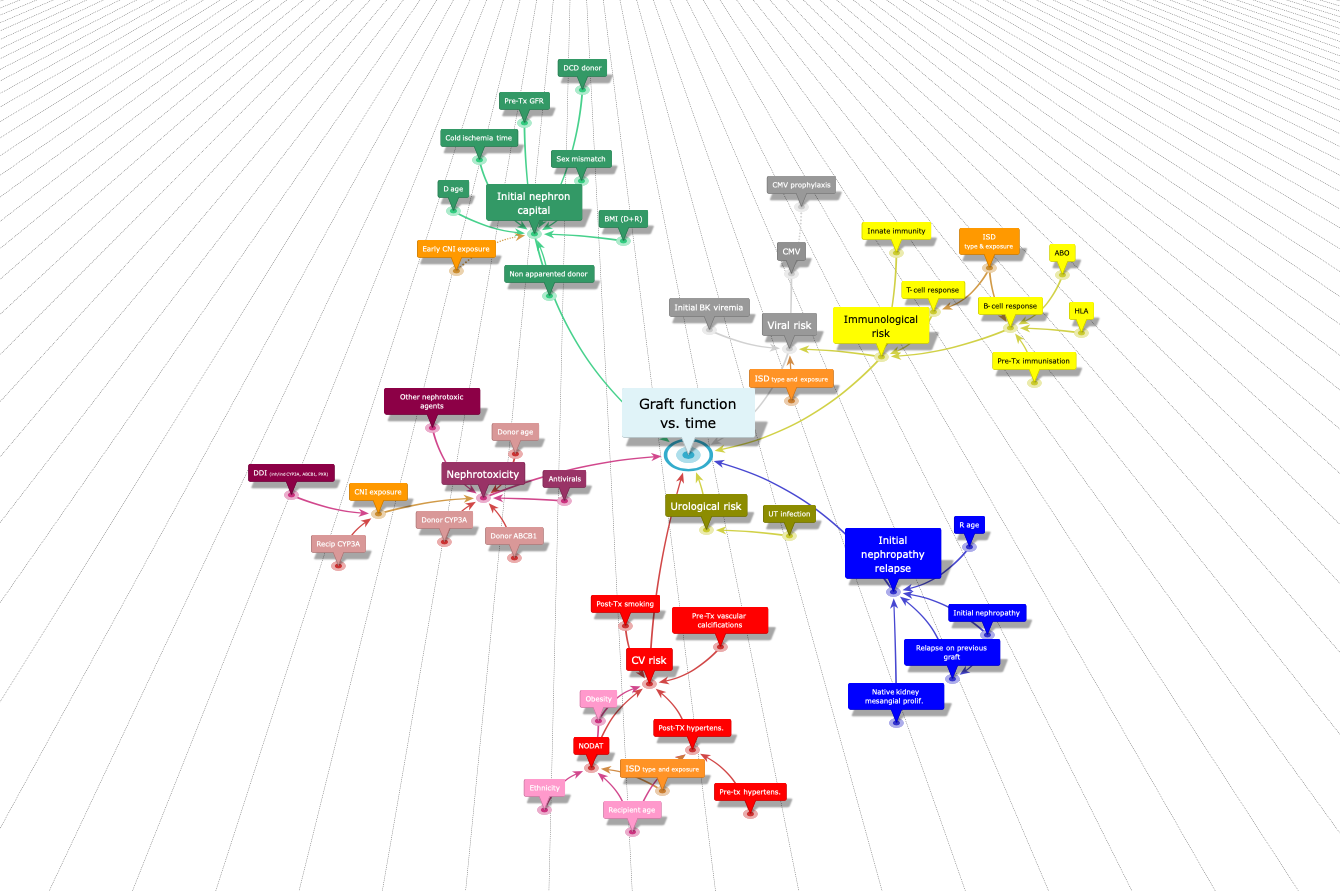
**Supplemental figure**

**Supplemental Figure 1:** Knowledge graph of the risks of kidney graft function decline over time
